# Implementing Remote Developmental Research: A Case Study of a Randomized Controlled Trial Language Intervention During COVID-19

**DOI:** 10.3389/fpsyg.2021.734375

**Published:** 2022-01-07

**Authors:** Ola Ozernov-Palchik, Halie A. Olson, Xochitl M. Arechiga, Hope Kentala, Jovita L. Solorio-Fielder, Kimberly L. Wang, Yesi Camacho Torres, Natalie D. Gardino, Jeff R. Dieffenbach, John D. E. Gabrieli

**Affiliations:** ^1^Brain and Cognitive Sciences, Massachusetts Institute of Technology, Cambridge, MA, United States; ^2^Harvard Graduate School of Education, Cambridge, MA, United States

**Keywords:** online studies, RCT, intervention research, developmental psychology, diversity

## Abstract

Intervention studies with developmental samples are difficult to implement, in particular when targeting demographically diverse communities. Online studies have the potential to examine the efficacy of highly scalable interventions aimed at enhancing development, and to address some of the barriers faced by underrepresented communities for participating in developmental research. During the COVID-19 pandemic, we executed a fully remote randomized controlled trial (RCT) language intervention with third and fourth grade students (*N* = 255; age range 8.19–10.72 years, mean = 9.41, SD = 0.52) from diverse backgrounds across the United States. Using this as a case study, we discuss both challenges and solutions to conducting an intensive online intervention through the various phases of the study, including recruitment, data collection, and fidelity of intervention implementation. We provide comprehensive suggestions and takeaways, and conclude by summarizing some important tradeoffs for researchers interested in carrying out such studies.

## Introduction

### Intervention Research in Developmental Science

One overarching goal of developmental research is to improve children’s outcomes. The most direct way to achieve this goal is to implement an intervention – some manipulation of a child’s experience or environment – and determine whether it leads to positive changes in outcomes. Not only do such studies allow researchers to test the efficacy of specific intervention programs, but they also play a crucial role in understanding developmental phenomena by elucidating causal mechanisms. A randomized controlled trial (RCT) design is a gold standard for establishing causality and efficacy in intervention research.

Despite the importance of intervention studies in developmental science, executing these studies is difficult. Because effect sizes tend to be small in developmental intervention studies, large samples are needed to detect significant effects (Lortie-Forgues and Inglis, 2019; Kraft, 2020). Interventions must be administered with high fidelity, which can be challenging at a large scale and when they require the involvement of caregivers or educators (Fixen et al., 2005; O’Donnell, 2008; Barton and Fettig, 2013). While in-lab intervention studies allow for highly controlled testing environments, they run the risk of not generalizing to real-world settings (Lortie-Forgues and Inglis, 2019). Additionally, in order to substantially impact a child’s experiences or environment, interventions typically have to be implemented over a long period of time (e.g., on the order of weeks to months). Both recruitment and retention of participants in developmental research intervention studies pose significant challenges.

Further, if interventions are to be translated into wide use, they have to be highly scalable to large numbers of children in diverse environments. In particular, the field of developmental research has recently come under scrutiny for predominantly studying WEIRD (western, educated, industrialized, rich, and democratic) populations (Nielsen et al., 2017). Even in the limited context of the United States, participants from lower socioeconomic status (SES) backgrounds are consistently underrepresented in research (Manz et al., 2010; Nicholson et al., 2011), and the majority of developmental science publications do not achieve a race/ethnicity distribution that matches that of the United States population (Bornstein et al., 2013). In addition to the profound issues related to equity (Lorenc et al., 2013; Veinot et al., 2018), lack of diversity and representativeness in developmental science threatens the generalizability of findings and fundamentally hinders our understanding of human development (Nielsen et al., 2017).

One major roadblock to the inclusion of more representative samples is the low participation rates of families from disadvantaged backgrounds in research (Heinrichs et al., 2005). There are multiple barriers to research participation that these families face, including informational barriers (not knowing about research opportunities), perceptual barriers (how families view the purpose and significance of research), and practical barriers such as lack of time and access to transportation (Heinrichs et al., 2005; Whittaker and Cowley, 2012). There are also many hard-to-reach communities in remote areas, far from universities and research centers. Practical barriers are most prohibitive for families from disadvantaged backgrounds (Lingwood et al., 2020).

### Online Studies: New Opportunities for Developmental Intervention Research

Online developmental research studies are becoming increasingly popular and have advanced rapidly during the COVID-19 pandemic. The main benefit of online studies is that they allow families to participate in research from the convenience of their own homes. These studies can take multiple forms, including moderated/synchronous video-based studies (i.e., a live experimenter interacts with a child over a video conferencing platform, such as the Parent and Researcher Collaborative^1^; see a review by Chuey et al., 2021), unmoderated/asynchronous video-based studies (i.e., through platforms that collect video without a live experimenter present, such as Lookit^2^; Scott and Schulz, 2017; for review see Rhodes et al., 2020), and unmoderated app-based studies (Gillen et al., 2021). Despite the increasing popularity of online developmental research and the promise of these methods for increased diversity and scalability (Casler et al., 2013; Scott et al., 2017; Kizilcec et al., 2020; Rhodes et al., 2020), online intervention research is still very limited (but see Kizilcec et al., 2020 for an example).

There are multiple factors to weigh when deciding whether and how to implement an online intervention study. For example, moderated research studies – particularly ones that target underrepresented populations – require a large investment of resources and labor (Rhodes et al., 2020). Using an online platform may increase geographic and racial representation (Scott et al., 2017; Rhodes et al., 2020), but at a potential risk of excluding low-income participants due to a lack of reliable internet and technology (Lourenco and Tasimi, 2020; Van Dijk, 2020). Disparities in access to internet and devices – i.e., the “digital divide” (Van Dijk, 2020) – were particularly apparent early in the pandemic, and concerns were raised about whether online studies would inadvertently decrease diversity in developmental studies (Lourenco and Tasimi, 2020). Finally, implementing research studies in participants’ homes, unlike in-lab studies, requires giving up some control over the study environment. In this paper, we describe some of the important factors to consider in the context of our experience implementing an intensive, fully remote RCT language intervention with third and fourth grade students (ages 8–10 years) from diverse backgrounds across the United States from summer 2020 – spring 2021. Notably, this study used a moderated online study design with extensive direct communication, and thus our suggestions are specific to this particular approach. We conclude by highlighting three main tradeoffs to think about when designing a remote intervention study with a developmental sample.

### Case Study: A Remote Language Intervention Study During the COVID-19 Pandemic

During the COVID-19 pandemic, we implemented an RCT intervention to assess the impact of listening to audiobooks on reading and language skills. Third and fourth grade students were randomly assigned to the Scaffolding, Audiobooks-only, or Mindfulness (active control) group. Children in the Audiobooks-only condition received unlimited access to audiobooks *via* the Learning Ally platform^3^, curated based on their listening comprehension level. Children in the Scaffolding condition also received audiobooks and recommendations, as well as one-on-one online sessions with a learning facilitator twice per week, focused on improving their listening comprehension strategies and supporting their intervention adherence. The Mindfulness group completed a control intervention using a mindfulness app. The intervention period was 8 weeks for each group, with 2–3 h of pre-testing and 2–3 h of post-testing using a battery of measures administered *via* Zoom. We believe that this project will serve as an informative case study for other developmental researchers considering adapting intensive developmental interventions to an online format. Hypotheses, detailed methods, and results from the study will be presented in a separate manuscript (Olson et al., in preparation^4^).

## Recruitment

An important consideration for developmental researchers planning an online intervention study is whether they will be able to recruit a large enough sample size within a feasible time frame. Furthermore, researchers may be looking to recruit samples that are representative in terms of demographic variables like race/ethnicity and SES. As a case study, we will first describe our final sample characteristics, and then outline specific examples of recruitment efforts throughout the study period that led to this sample, including costs for various recruitment strategies.

### Participants

Beginning in mid-summer 2020, we set out to recruit 240 third and fourth grade students (80 per group) with a broad range of demographic, geographic, reading level, and SES characteristics. To be eligible for the first pre-testing session, children had to be fluent in English, have a caregiver who spoke English or Spanish, and have no diagnosis of autism spectrum disorders or hearing impairments. Given that all sessions were held virtually, over Zoom, we unfortunately could not accommodate families who did not have internet or computer/tablet access (*N* = 14). However, because this study took place during the pandemic, many school systems provided children with access to these resources. We reached back out to families who expressed interest but initially lacked a computer and/or internet over the summer to see if they had been provided these resources by the school system during the school year. Since many families in poor and rural communities lack access to reliable internet (Lourenco and Tasimi, 2020; Van Dijk, 2020), our sample may not be representative of the most severely affected lower-income communities. Children were compensated $20 per hour for all pre-testing and post-testing sessions (approximately 6 h total during the study). Caregivers were additionally compensated $5 per survey for completing a total of ten surveys at the beginning and end of the study. Families also received lifetime access to the Learning Ally audiobook service after completion of the study, regardless of their group assignment.

**FIGURE 1:**
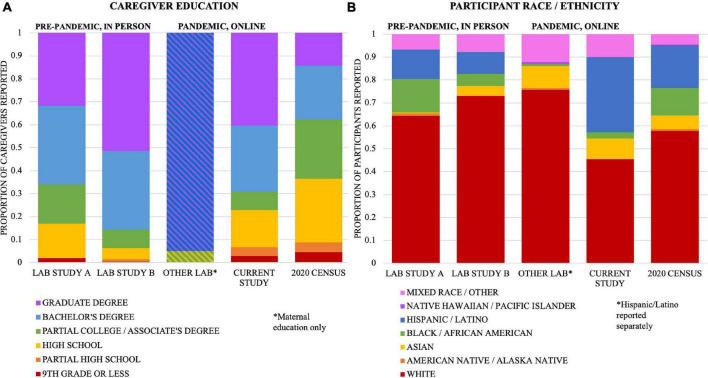
Comparison to two studies from our lab conducted prior to the pandemic (Lab Study A, Ozernov-Palchik et al., 2017; Lab Study B, Pollack et al., 2021), one from another lab conducting a similar study during the pandemic (Other Lab; Bambha and Casasola, 2021), and the 2020 US Census. For Lab Study B, we included all participants who completed any portion of the study. **(A)** Highest level of parental education attainment, including both parents, for all who responded (Lab Study A, *N* = 358; Lab Study B, *N* = 463; Other Lab [maternal only], *N* = 118; Current Study, *N* = 449). 2020 Census includes all adults 25 years and older. **(B)** Parent-reported race/ethnicity of the child, for all who responded (Lab Study A, *N* = 179; Lab Study B, *N* = 230; Other Lab, *N* = 115; Current Study, *N* = 231). Participants who identify as Hispanic/Latino are counted in that category, regardless of race. Other categories reflect that race alone (not Hispanic/Latino). *Bambha and Casasola reported maternal education only: obtained high school degree (118/118), obtained 4-year college degree or above (112/118); and reported Hispanic/Latino separately from race (15/115 were Hispanic or Latino).

Figure 1 shows demographic information for the 255 participants (age range 8.19–10.72 years, mean = 9.41, SD = 0.52) who were eligible for our study and were included in one of our three intervention groups, as well as how our sample compares to the United States Census data from 2020 (excludes participants who did not respond to these questions; NA = 24 for race/ethnicity, NA = 24 for maternal education, NA = 37 for paternal education). To demonstrate how the sample demographics in this study compare to similar in-lab and online studies, we also show demographic distributions from three comparison studies (Table 1 and Figure 1): a pre-pandemic longitudinal neuroimaging study conducted in our lab that relied on school partnerships and in-school testing for recruitment (Lab Study A, Ozernov-Palchik et al., 2017), a neuroimaging study conducted in our lab that used a combination of outreach events, advertisements, and social media to recruit participants (Lab Study B, Pollack et al., 2021), and an online intervention study conducted by another lab during the pandemic (Other Lab, Bambha and Casasola, 2021). We conducted a chi-square analysis to compare differences in the frequency of children with parental education of only high school between the current study and the four comparison samples (i.e., Lab Study A, Lab Study B, Other Lab, Census). The current study was not significantly different in the frequency of high school level education or below than the Lab Study A [*X*^2^(1) = 3.12, *p* = 0.078] and Lab Study B [*X*^2^(1) = 0.3, *p* = 0.584], but it had higher frequency of high school level education or below than the Other Lab study [*X*^2^(1) = 26.15, *p* < 0.001] and lower frequency than the 2020 United States Census data [*X*^2^(1) = 76.6, *p* < 0.001]. For a study conducted entirely online and during the pandemic, we successfully achieved a socioeconomically diverse sample comparable to pre-pandemic in-person studies that relied on in-school recruitment. Notably, the comparison online study – which did not specifically aim to recruit a diverse sample in terms of SES – included almost all mothers with at least a 4-year college degree. Thus, the transition to online studies does not automatically increase participant diversity in terms of SES.

**TABLE 1:** Comparison to three representative studies.

Study	*N*	Age range	Setting	Recruitment	Time	Type
Lab Study A	182	8–10 years	Lab	School partnership	Pre-pandemic	Neuroimaging/longitudinal
Lab Study B	248	8–13 years	Lab	School outreach + social media	Pre-pandemic	Neuroimaging
Other Lab	118	3–5 years	Online	Social media	Pandemic	Intervention
Current study	255	8–10 years	Online	School outreach + social media	Pandemic	Intervention

**FIGURE 2:**
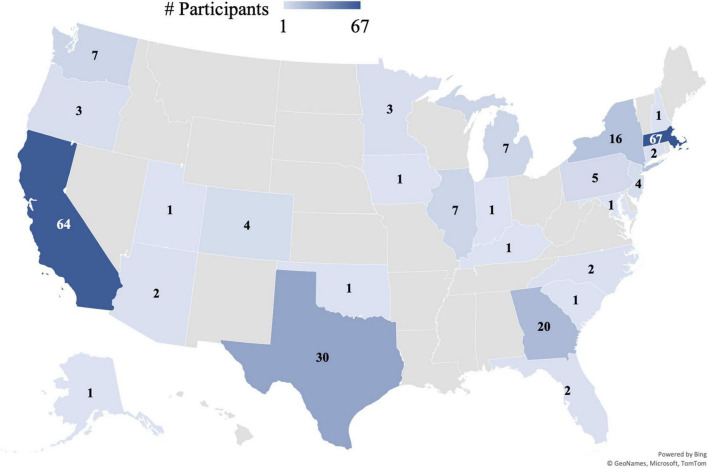
Map of participants by state. Map shows number of participants per state that were sorted into one of the three intervention groups (*N* = 255). Not shown: 1 participant from Canada.

We also evaluated differences in the frequency of white participants across the five samples. Our study had a lower frequency of white participants than Lab Study A [*X*^2^(1) = 13.58, *p* < 0.001], Lab Study B [*X*^2^(1) = 35.19, *p* < 0.001], the Other Lab study [*X*^2^(1) = 27.14, *p* < 0.001], and the 2020 United States Census [*X*^2^(1) = 14.02, *p* < 0.001]. The majority of developmental studies do not have representative samples in terms of racial diversity (Bornstein et al., 2013). There are important caveats to the comparison between the current study and the other lab studies, however. The in-lab studies were not conducted during a pandemic, and they involved neuroimaging. Despite their longitudinal nature, the in-lab studies did not include an intervention, which may have incentivized participation from some families. Nevertheless, although the comparison is not well-controlled, it suggests that we were successful in recruiting a diverse, representative sample of participants. Furthermore, we attained substantially more geographic diversity than is possible with in-lab studies. Our 255 participants came from a total of 26 states and 186 zip codes in the United States, plus Canada (Figure 2).

### Overall Recruitment Strategies

To attain a diverse sample for our online intervention study, we tried several avenues for recruitment, including existing relationships with schools, new school partnerships, and online advertising. We received MIT Institutional Review Board (IRB) approval for all of our recruitment materials including flyers and social media ads in English and Spanish. These flyers and ads included a link directing caregivers to our participant screening survey. All study data, including data from the screening survey, were managed using REDCap (Research Electronic Data Capture), a secure, web-based software platform designed to support data capture for research studies (Harris et al., 2009, 2019). The landing page, available in English and Spanish, briefly outlined the study and asked the parent or guardian to provide contact information, a simple demographic profile of their child, and other factors relevant to the study (e.g., access to technology). We included the question, “Does your child receive free or reduced lunch at school?” and prioritized contacting the families that responded ‘yes’ to this question. Below, we describe the efficacy of our different recruitment strategies, as well as our takeaways for other researchers considering these methods for an online intervention study.

#### School Partnerships

**FIGURE 3:**
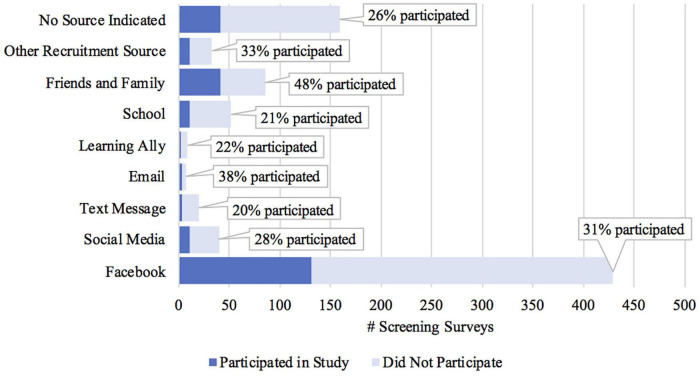
Completed screening surveys and final participants by recruiting source.

We began recruitment efforts in summer 2020 by reaching out to large and diverse school districts with whom we had existing relationships. Our hope had been to disproportionately recruit lower SES students based on the profiles of the districts, such as public schools with high percentages of free/reduced lunch eligibility. We met with district leaders and principals, who expressed their enthusiasm and commitment to supporting our study. Fourteen schools, all with a large proportion of free/reduced lunch eligible families, officially partnered with our study. Outreach efforts by educators at our partner schools included pre-recorded phone calls to families, flyers, and text messages, with a range of 3–8 outreach attempts per school to their eligible students. This outreach yielded a relatively small fraction of the target number of students (Figure 3). It is important to note, however, that our school recruitment efforts took place during the early months of the pandemic when many educators were managing the logistics of school closures, and caregivers were getting accustomed to the new realities of remote learning. Additionally, our school partnership efforts were limited to schools with predominantly English and Spanish speaking parents and caregivers, as we were not able to accommodate families in additional languages. Online intervention studies that choose to focus their recruitment on specific school districts should likewise consider the predominant language(s) spoken within the community, as we found that our study required substantial ongoing communication with families to provide appropriate support and ensure adherence (see “Family Communication and Retention” section, below).

#### Social Media

Our biggest recruitment success came from social media advertising through Facebook and Twitter. However, recruiting *via* these modalities introduced a unique set of challenges and considerations. One other online option we pursued was Craigslist targeted for specific zip codes, but this approach was ineffective due to Craigslist’s stringent policies regarding the categorization of ads.

#### Facebook

We first posted about our study on our lab’s Facebook page. Our lab had existing relationships with parent advocacy groups and other organizations that serve students with language-based learning disabilities. These organizations were more likely to include families from higher-SES backgrounds, so our initial social media recruitment efforts were skewed toward this demographic. We then transitioned to paid Facebook ads. Our initial push was not as fruitful, primarily due to a low budget: we originally invested $25 per posted ad, with each post spanning 3–5 consecutive days within a week. Each week, we launched a different ad until we exhausted our three differently themed ads (each available in English and Spanish), then started the sequence over again. After a month, we increased the budget to $300 per posted ad for subsequent weeks. With this latter approach, we settled on three consecutive 24-h days, usually Friday–Monday. Table 2 summarizes Facebook ad effectiveness for different representative configurations of ads.

**TABLE 2:** Effectiveness for three representative Facebook Ad configurations.

Ad configuration	Total spend	Impressions	Clicks	Clicks per thousand impressions	Cost per click	Cost per participant
**Set A: 25-mile radius around select cities**						
English Ads	$1,714	273,448	3,030	11.1	$0.57	n/a
Spanish Ads	$363	78,593	709	9.0	$0.51	n/a
**English + Spanish Ads**	**$2,077**	**352,041**	**3,739**	**10.6**	**$0.56**	**$17.02**
**Set B: 10-mile radius around select cities**						
English Ads	$1,089	160,038	1,823	11.4	$0.60	n/a
Spanish Ads	$373	61,952	524	8.5	$0.71	n/a
**English + Spanish Ads**	**$1,463**	**221,990**	**2,347**	**10.6**	**$0.62**	**$86.05**
**Set C: low SES zip codes**						
English Ads	$579	99,180	579	5.8	$1.00	n/a
Spanish Ads	$271	37,984	212	5.6	$1.28	n/a
**English + Spanish Ads**	**$849**	**137,164**	**791**	**5.8**	**$1.07**	**$283.06**
**TOTAL**	**$4,389**	**711,195**	**6,877**	**9.7**	**$0.64**	**$33.50**

*Total spend, number of advertisement impressions, number of clicks on our screening survey, number of clicks on our screening survey per thousand ad impressions, and the cost per click on our screening survey are shown for three of our Facebook advertisement campaigns. Estimated cost per participant was calculated based on participant report of how they found out about our study on the screening survey (N = 255 total participants began the intervention).*

Not surprisingly, it quickly became apparent that the amount of money invested resulted in increased study interest; the higher the investment, the more the ad is advertised across Facebook, Instagram, and Facebook messenger. The more the post is advertised, the greater the opportunity for engagement, and ultimately increased participation numbers. For future studies, if using Facebook, we recommend a generous social media budget to yield a large pool of participants. In total, we spent $4,389 on Facebook advertisements over the course of the study, and a total of 131 of our 255 participants indicated that they found out about our study *via* Facebook (Figure 3), resulting in an average cost of approximately $34 per participant recruited *via* Facebook (Table 2). However, the actual cost per Facebook-recruited participant varied widely during different ad campaigns (Table 2).

To help us recruit participants from lower-SES backgrounds, we used targeted advertising. Facebook provides an option to target specific audiences by selecting cities, zip codes, educational level, age of child, individual interests, and more. While more individuals from targeted communities will see the post across their social media accounts, it does not necessarily mean that each individual who engages with the post will enroll in the study, so consistently posting is key to increasing enrollment rates. For instance, after boosting our recruitment success by targeting ads at 25-mile radius circles around select cities (variously, Atlanta, Boston, Chicago, Dallas, Detroit, Houston, Los Angeles, Miami, New York, Philadelphia, Phoenix, and San Antonio), to target families closer to urban centers, we narrowed the radius to 10 miles in an attempt to recruit more lower-SES participants. Recruiting to this profile proved less successful than it was for the 25-mile radius group. We then used a “household income by zip code” list to try to further improve lower-SES recruitment, but as with the 10-mile radius effort, this approach was not successful. Table 2 shows estimated costs per participant (qualified and began the intervention) who learned about our study from one of the three ad campaigns. It should be noted that these estimates rely on open-ended report of how participants learned about the study, and that these ad campaigns proceeded sequentially over different times during the year, with substantial variation in exactly which areas were targeted. Thus, while we think the estimates are informative for researchers considering these strategies, many factors likely influenced the number of participants we recruited.

#### Twitter

Learning Ally, the non-profit audiobook company that we partnered with for the study, advertised our study *via* Twitter (Table 3). We attribute their much higher ad engagement (about 10x what we saw with Facebook) to their large and strong following. This higher ad engagement did not translate to more sign-ups, however, as no participants explicitly identified Twitter as how they found out about our study.

**TABLE 3:** Effectiveness for Twitter Ads.

Ad configuration	Total spend	Impressions	Clicks	Clicks per thousand impressions	Cost per click
Total campaign	∼$450	∼20,000	1,793	91.0	$0.25

### Takeaways

School partnerships allow for greater control over participant demographics, as researchers can choose to partner with schools that have specific demographic profiles. However, establishing these partnerships takes time and effort, and may yield modest recruitment for an intensive, out-of-school intervention program. While it is certainly possible to establish school partnerships for an online intervention study, it does require substantial resources (both time and money) from the research team. Social media advertising brings the benefits of both large reach and precision targeting. Since online intervention studies do not have geographic constraints, this recruitment strategy may be beneficial for other developmental researchers considering implementing an online intervention.

Response rates per ad shown are quite small – close to 800,000 people viewing the ad yielded less than 150 actual participants. For the paid advertising, the cost ranged from $0.25 to $1.40 per ad click. This relatively wide difference reflects whether the audience knows the advertiser (in the case of Learning Ally’s Twitter audience), how the ads were targeted by SES level (lower SES clicks had a higher cost), and what language the ads were in (English had a lower cost than Spanish). It is important to note that clicks do not remotely equate directly to study participants – the vast majority of people reaching the screener landing page (95%+) did not sign up for the study.

Overall, our recruitment efforts led to a representative sample of participants in terms of caregiver education and child’s race/ethnicity (Figure 1). We also attained substantial geographic diversity, with participants from 186 different zip codes and 26 different states in the United States, plus Canada (Figure 2). Our sample was not substantially more diverse in terms of caregiver education compared to other studies run by our lab that aimed to recruit diverse samples, but it was more diverse than another similar study run during the pandemic that did not explicitly aim to recruit a diverse sample based on caregiver education. Our sample was also more ethnically/racially diverse than similar in-lab studies and the general United States population. Thus, the transition to an online intervention format does not necessarily lead to more diverse samples on all dimensions without explicit efforts on those fronts, as well as a considerable recruitment budget.

## Family Communication and Retention

Another factor developmental researchers will need to consider when adapting to an online protocol for intervention studies is how to ensure continued engagement and adherence to the program. During our study, not only were we collecting data and administering an intervention online, but we were also doing so during a global pandemic. Families dealt with illness, death, financial stress, technological challenges, and other difficulties over the course of the study. We adapted our communication protocols to be as supportive to families as possible. We believe that these lessons are also worth sharing, as even in non-pandemic times, families encounter these and other challenges.

### Personalized Communication Methods

Having robust procedures for family scheduling and communication was vital to our study. We had a dedicated research team whose primary role was to contact families and answer any questions that came up. This team included two full-time research staff, as well as 2–3 undergraduate research assistants available to troubleshoot specific questions regarding the use of the audiobook app. We received 15–35 emails per day regarding scheduling, rescheduling, payment requests, score report updates, app issues, etc.

**FIGURE 4:**
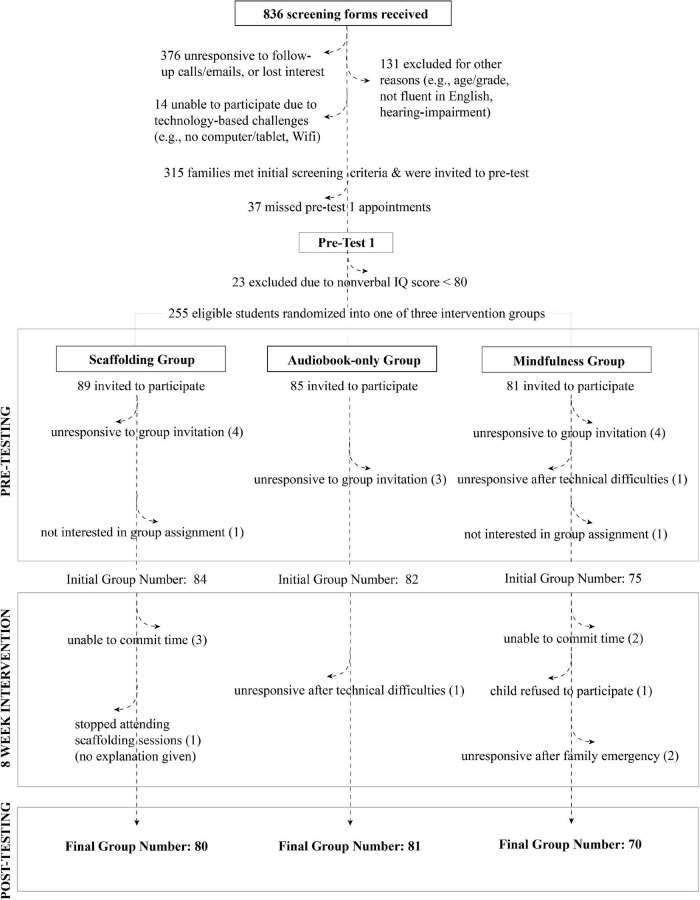
Participant pipeline and attrition.

Before the study began, we drafted email and text templates for key communication points at various stages before, during, and after the intervention. For example, we had templates for program orientation and onboarding procedures, appointment confirmation and session reminders, as well as periodic check-ins. In our screening form, we asked for each family’s preferred method of communication, and we used this method throughout the study. To ensure consistency in communication, one researcher was assigned to each family and handled all communication for that family. While communicating with families using various methods (i.e., emails, phone calls, text messages) was more time and labor intensive, we found that it boosted participation throughout the duration of our study. We observed high retention rates overall, but there was still attrition (Figure 4). Text and email reminders helped minimize missed appointments. If participants missed a session or were generally more challenging to communicate with, we noted this for their next session and asked the tester to send an additional reminder the day of the testing session to ensure attendance.

For the Scaffolding Group in our study (i.e., the intervention group that met biweekly with ‘learning facilitators’ in addition to listening to audiobooks), the average family required approximately 37 points of contact throughout the study. This included appointment confirmations, reminders about reading books, payment details, and parent surveys. Similar levels of communication were required for the other groups (i.e., Audiobook-only and Mindfulness), with around 24 points of contact per family. Importantly, however, the number of contact points per family within each group varied based on families’ circumstances. Families with limited access to and knowledge of technology at home required additional support throughout the study from our research team. Families with more variable work schedules were more likely to miss sessions or need to reschedule. Thus, we strongly advocate for clear, consistent, and individualized communication with all families, which may especially affect the enrollment and retention of the participants from disadvantaged backgrounds.

#### Importance of Bilingual Research Personnel

There was a large proportion of Spanish-speaking families in our partner schools. Our final sample included 12% Spanish-speaking participants (30/255), and we had two bilingual Spanish-speaking full-time researchers to support these families. At the beginning of the study, there was a large effort to translate all study materials, surveys, and additional resources into Spanish. Although most of the translation effort was front-loaded, there was still a need for Spanish-speaking researchers throughout the study for family communication.

#### Scheduling

Since we had families from across the United States participate in our study, we had to account for multiple time zones when scheduling sessions with testers and learning facilitators. We were able to schedule sessions around each family’s schedule, including weekend and evening sessions. Each tester had a personal, secure Zoom link that was sent to the family before their scheduled session. Unlike in-person data collection, there was no limit to how many sessions we could book at one time, since physical space was not an issue. Testers called and attempted to troubleshoot with the family if the participant had difficulty getting onto Zoom. The child could complete sessions on a computer or tablet; we also allowed children to log onto the Zoom session *via* a cell phone in circumstances where no other option was available (only for tests without visual stimuli, as image size would be significantly reduced on a phone screen).

### Retention

Most families who expressed initial interest by filling out our screening survey did not end up participating in our study. We experienced high attrition between screening, pre-testing, and group assignment. However, once participants completed onboarding procedures and began the 8-week intervention, attrition was quite low (Figure 4).

Flexible accommodations to families’ individual needs minimized mid-study attrition, but it could not be entirely prevented. In some cases, children were very resistant to participating in the intervention. For example, given the new distance-learning protocols implemented during the COVID-19 pandemic, some children reported not wishing to have more screen-time. This may be relevant for future studies if educators continue to rely on screen-based technology for learning in and out of school. In instances where children were especially resistant to participating, we did not pressure them to continue. In other instances, however, families simply stopped responding to emails and texts. We observed the greatest non-responsiveness at the end of the program, when attempting to schedule post-test sessions. When faced with non-responsive participants, we first followed-up with multiple (∼5) reminder emails, phone calls, and/or text messages, then we issued one final check-in email before suspending any further attempts to reach out.

### Takeaways

Overall, we credit the efficiency of our communication pipeline to the use of pre-drafted email/text templates and maintaining an active log of all communications. We recommend frequent and consistent communication with participants to minimize attrition when conducting large-scale online intervention studies. Being timely with responses encourages participants to continue with the study and increases their participation at the post-testing portion of the study. To manage a large number of participant questions, it is important to have a main contact person for each family. We found that regular interaction with our families, *via* their preferred mode of communication, was effective in establishing rapport and maximizing engagement. Moreover, it is critically important to have bilingual staff who can closely support families who may speak another language. Finally, detailed study orientation materials and clear, step-by-step onboarding procedures are useful to ensure that participants understand all study requirements and to preemptively troubleshoot potential barriers to participation.

## Data Collection

For developmental researchers that typically utilize in-lab assessments, a major adjustment when transitioning to online intervention studies is adapting measures for online administration. Here, we describe the measures we used, how assessment scores compared to in-lab administration of the same assessments, how we dealt with variable testing environments, and how we trained our team to administer assessments online.

### Behavioral Battery Adaptation

Adapting assessments for online administration required careful consideration to ensure feasibility for both testers and participants. We decided to administer all assessments over Zoom, which allowed testers to directly interact with participants in real-time. For scoring purposes, we audio- and video-recorded each session and stored these recordings securely. The Zoom platform enabled testers to share their screens, allowing us to display scans of stimulus items and online assessment platforms.

Online administration of the assessments in our battery required various considerations and adaptations (Table 4). Some tests had already been adapted for online administration, and we used the publisher’s online administration and scoring platform. Other tests required tracking the child’s responses and simultaneous scoring that was not viable *via* the computer. We mailed packets to each tester containing printouts of these assessment score sheets along with dry-erase markers and plastic protector sheets. This packet also included a copy of the testing manual containing the required materials and procedures for all tests. These materials allowed testers to have fewer files open on their computer at once during the session. We used DropBox (MIT provides large storage space to its affiliates) to upload all materials for tester access (e.g., stimulus item scans, administration guidelines, etc.), and we used team Slack as a way to troubleshoot or to ask questions before, during, or after test administration.

**TABLE 4:** Assessments and adaptations for remote administration.

Assessment	Description	Adaptations	Sample reliability coefficients	Publisher reliability coefficients
Kaufman Brief Intelligence Test, 2nd Edition (KBIT-2) – Matrices^1^	Standardized non-verbal IQ assessment	Scan of stimulus items screen-shared *via* Zoom	α: 0.83 Split-half: 0.81	Split-half: 0.81–0.88
Clinical Evaluation of Language Fundamentals, 5th Edition (CELF-5) – Understanding Spoken Paragraphs^2^	Standardized test of listening comprehension	Administered *via* Zoom	α: 0.74 Split-half: 0.79	α: 0.75–0.85
Dynamic Indicators of Basic Early Literacy Skills (DIBELS)^3^ • Word Reading Fluency (WRF) • Passage Reading Fluency (PRF) • Multiple Choice Reading Comprehension (MCRC)	Standardized measures to assess reading skills; MCRC is a computer-administered standardized test	WRF and PRF: Digital forms screen-shared *via* Zoom. Tester recorded errors on online progress monitoring site from publisher. MCRC: Tester screen-shared and child was given control of tester’s screen to select multiple choice answers. Alternative was to have child orally tell tester which answer to select (when child was unable to utilize “Remote Control”).	Item level data was not available	
Peabody Picture Vocabulary Test, 5th Edition (PPVT-5)^4^	Standardized receptive vocabulary assessment	Images screen-shared *via* Zoom using publisher materials adapted for digital use (Q Global).	α: 0.96 Split-half: 0.96	α: 0.97
Wechsler Abbreviated Scale of Intelligence, 2nd Edition (WASI-II) – Vocabulary^5^	Standardized vocabulary assessment	Scan of stimulus items screen-shared *via* Zoom.	α: 0.8 Split-half: 0.82	Split-half: 0.88–0.93
Comprehensive Test of Phonological Processing, 2nd Edition (CTOPP-2) – Non-word Repetition, Memory for Digits, Blending Words^6^	Standardized measures to assess baseline working memory skills	Audio files sent to families to download ahead of time; child/caregiver asked to play each file from their computer during assessment.	NWR α: 0.73 Split-half: 0.76 MD α: 0.8 Split-half: 0.84 BW α: 0.84 Split-half: 0.86	α: 0.77 α: 0.8 α: 0.8

*^1^Kaufman, 2004; ^2^Wiig et al., 2013; ^3^Good et al., 2002; ^4^Dunn and Dunn, 2007; ^5^Wechsler, 2011; ^6^Wagner et al., 1999.*

*α represents the Cronbach’s alpha and split-half represents the Spearman–Brown prophecy formula. Reliability coefficient values above 0.71 are considered acceptable (George and Mallery, 2003). Publisher reliability information was obtained from the technical manuals and reports released by the respective companies.*

### Tester Training

The testing team consisted of graduate students from speech and language pathology or early education programs with experience administering psychoeducational evaluations to school-aged children. All testers were native English speakers, and some were also fluent in Spanish. All testers had prior knowledge of the Zoom platform and different file storing/sharing programs (e.g., DropBox, Google Drive). Testers were trained remotely on administering and scoring our assessment battery. Before starting their first session, testers scored a video-recorded session and were deemed ready if they achieved 95% reliability with the first scorer (an experienced tester). A team member reviewed and scored the video recording of each tester’s first session with a child and gave them feedback as necessary. Training continued until the testers were able to administer and score all assessments with high accuracy. Testers were blind to participants’ group assignments. One benefit of online testing is the ability to easily video record testing sessions. Doing so helped facilitate a more thorough reliability assurance than for in-lab studies that tend to only audio-record sessions.

### Remote Administration

We also needed to adapt our general assessment administration procedures. Each session began with the tester confirming the child was in an optimal testing environment, and adjustments were made if necessary (i.e., moving to a quieter space in the home). Caregivers were asked for their permission to have the Zoom session recorded. The tester then reviewed the consent form with the caregiver and the assent form with the child, which had been emailed to the family before the session, and obtained verbal consent from both the caregiver and child. If the family’s primary language was Spanish, the initial session was scheduled with a bilingual tester, or another bilingual member of the team joined the session to obtain consent in Spanish. Testers then administered the assessments. These were split across 2–3 sessions, as the battery of tests was extensive, and children generally fatigued after about 90 min. Immediately following the session, testers uploaded the recordings of both the verbal consent/assent and the testing session to a secure server, and submitted records of participants’ responses.

Finally, we needed to establish data management and scoring procedures that ensured accuracy in the online setting. Since paper record forms could not be centrally stored with all of our testers working remotely, we created Google Forms to record participants’ responses for most assessments. Having digital copies of item-level responses helped with easily calculating reliability for each assessment (Table 4). The Google Forms were used to generate spreadsheets of participant data for each assessment. All records only used participant IDs. Other assessments required the use of the developer’s platform for scoring.

#### Testing Environment

The testing team encountered a variety of challenges unique to the virtual testing environment. In-person assessment allows for more knowledge of and control over what participants are doing during the session. With online administration, we relied more on children and caregivers to achieve consistency in the testing environment. For instance, during the online sessions, we needed to make sure that participants could see and hear what we expected them to, despite not having direct control over the visual display and audio output of their devices. Thus, testers regularly asked participants to confirm that they could see the screen-shared materials and hear their voice clearly, adjusting the size of materials on display and asking children to adjust their speaker/headphone volume as necessary.

The most common issues were loud background noise in the home and poor internet connection, which often affected audio quality for the participant, tester, or both. It was sometimes difficult to judge the quality of what the child was hearing, especially when caregivers were not present to provide feedback. For assessments that involved timed performance or stimulus items that could not be repeated, testers made adjustments to reduce validity concerns. If there was background noise and the child did not have a quieter space, testers asked the child to put on headphones or saved listening tasks for the following session when the child might be in a quieter environment. Child responses were often difficult to discern when answer choices involved rhyming letters (e.g., A, B, C, D), even after asking the child to repeat the response. In these instances, testers requested that the child type their answers into the chat on Zoom.

Internet connectivity and other technical factors (e.g., the ability to download and play audio files provided by the team) varied widely across participants and between sessions. Sometimes testers turned off the video portion of the Zoom call in an attempt to improve the audio connection. The team also encountered minor technical issues with specific aspects of the online administration process, such as problems with using the “Remote Control” function on Zoom on certain types of computers.

At times, the participant’s home environment was distracting for other reasons, such as family members or pets entering the room. Many children completed the testing from a desk, but many others completed it while sitting on a couch or in their bed, and some children needed reminders to sit up or change position to better focus on assessment tasks. Because some caregivers chose to remain in the room during testing, testers occasionally encountered caregivers who continued to help their child despite the tester’s requests not to. In particular, because caregivers were often off-camera, it was sometimes difficult to gauge the extent of the support given by the caregiver. The presence of caregivers in the room may have made some children more self-conscious about their performance, whereas other children appeared comforted by their presence. Also, because the tester could not see the child’s screen, some children may have attempted to look up answers to certain testing questions, though we do not believe this to be a significant issue overall. The ability to record and re-watch sessions while scoring was critical given these challenges unique to the home setting.

Finally, some children felt fatigued during sessions scheduled after the child had just spent several hours on the computer during remote learning. Testers offered breaks and/or ended the session based on their judgment of the child’s fatigue and engagement.

#### Scoring

To ensure validity, each assessment was double-scored by another tester. The second scorer watched session recordings (stored and accessed on a secure server) to verify the original scores provided by testers. If there were discrepancies between first and second scores, a core research team member who is an experienced clinician made the final scoring decision.

Scorers used an online spreadsheet to document the scoring process: the team would notate who second scored a test, their calculations of scores, any scoring discrepancies that were resolved, and any validity issues within a testing session. The scoring spreadsheet also contained formulas to automatically calculate raw scores to make the process more efficient. The second scorer documented the final scores in REDCap. Scorers were encouraged to consult and communicate with the team whenever scoring questions or concerns arose.

#### Reliability

We computed Cronbach’s alpha and split-half reliability for all of the standardized tasks administered in our study, except for one task where item-level information was not available from the publisher’s website (DIBELS). Table 4 provides reliability coefficients for the current study and, for comparison, the coefficients provided from the publisher for each of the subtests. The reliability coefficients for the online administration of the subtests were comparable to those reported by the publishers and are considered to be within the acceptable-good range.

##### Measurement Error

To further evaluate whether online administration of assessments introduced a measurement error, we calculated pairwise correlations among the standardized measures used in this study that overlapped with those administered for a different pre-pandemic in-person study in the lab (Lab Study A; Table 5). The comparison study (Lab Study A, Ozernov-Palchik et al., 2017) included 158 rising third-grade students with complete data for the relevant tests. Participants for this study were recruited from 21 schools in New England and represented a demographically similar sample to that of the current study (Figure 1). The correlation patterns among the variables in both studies were similar, suggesting that the same constructs were evaluated in the online version of the assessments as in the in-person version.

**TABLE 5:** Pairwise correlations among a sample of six variables from the current study and a comparable pre-pandemic in-person study from the same research lab.

Previous in-person sample of 3rd graders *N* = 158					
	PPVT	CELF	KBIT	Blending words	Memory for digits
CELF	0.56***				
KBIT	0.30***	0.23**			
Blending words	0.43***	0.39***	0.14		
Memory for digits	0.46***	0.35***	0.27***	0.46***	
Non-word repetition	0.56***	0.35***	0.15	0.57***	0.51***

**Current sample**					

	**PPVT**	**CELF**	**KBIT**	**Blending words**	**Memory for digits**

CELF	0.43***				
KBIT	0.49***	0.33***			
Blending words	0.40***	0.28***	0.22**		
Memory for digits	0.40***	0.23**	0.20**	0.29***	
Non-word repetition	0.37***	0.31***	0.24**	0.40***	0.37***

***p < 0.001, **p < 0.01.

### Takeaways

The training process for administration and scoring of online assessments was more labor intensive than in-person studies, as there was an additional layer of developing tester competency with managing Zoom, engaging the child, and recording scores in an accessible way. Difficulties included connectivity issues and controlling for the environment (i.e., background noise, distraction). The lack of control over the child’s home environment posed some reliability and validity concerns, but the flexibility of online administration also allowed for a greater ability to adapt to children’s and families’ individual needs. Some children may have benefited from testing in their home environment, as testing in an unfamiliar location can lead to anxiety or stress.

For those planning to implement an online testing battery in an intervention study, we recommend setting up clear and detailed systems for documentation. The amount of digital documentation was greatly increased through adaptation for virtual administration. Materials and data should be organized in the most centralized and streamlined way possible to avoid confusion and misplacement of files. Not all stimuli and record forms can be easily adapted for online administration, and alternative methods (e.g., scanning the original form) may need to be considered depending on the assessment and availability of technology to testers and participants. It can be helpful to compile a document outlining each test, how it is administered, and links to any websites or documents needed for administration so the team has a centralized procedural document to follow.

We also recommend that before starting a new online study, researchers outline guidelines for addressing technical or environmental issues that inevitably arise (e.g., what to do if you are having trouble discerning the child’s answer *via* Zoom). Technical and environmental factors cannot be eliminated when assessments are being administered virtually, but clear procedural guidance and detailed documentation during testing (e.g., noting the child’s behavior and any technical issues) can help reduce reliability and validity concerns. Having an online, real-time messaging system (e.g., Slack) is also an essential tool to ensure the team is able to communicate questions and concerns. Overall, the results for the standardized measures in the current study suggest equivalent effects of online testing to those of in-person testing, which are encouraging for the potential for future online intervention studies.

## Intervention

Developmental researchers transitioning to a fully online intervention study will need to carefully consider how to adapt materials, train the research team (particularly if they are not located in the same place), and address difficulties that may be more likely to arise in online settings. In particular, intensive implementation of an intervention during the pandemic introduced new challenges related to privacy and disclosure. Finally, qualitative data on individuals’ experiences participating in the study is important for identifying potential confounds and limitations, as well as for considering future scalability. We conclude this section by providing examples of feedback received from children and caregivers in our study.

### Curriculum Adaptation

In our study, the Scaffolding Group received biweekly scaffolding sessions led by learning facilitators. For these sessions, we adapted an existing curriculum targeting oral language skills in elementary school children developed by the Language and Reading Research Consortium (LAARC; Jiang and Logan, 2019). We added verbatim scripts for the learning facilitators to read to the children. Before each session, the learning facilitators adapted these scripts to the particular text they were working on with their child. The online format allowed learning facilitators to more easily follow a script than during face-to-face communication, thereby assuring greater fidelity of implementation. We also adapted materials that are designed for use by teachers in a physical classroom to online administration. For example, we used the whiteboard feature in Zoom to draw and write words during the lesson. As part of their preparation for each lesson, the learning facilitators prepared slides with pictures of vocabulary words from the books. We embedded explicit instructions on how and when to utilize these virtual materials for each strategy. To avoid boredom and distraction, we incorporated activities to optimize child engagement during each lesson. All scaffolding sessions were recorded and stored on a secure server.

### Learning Facilitator Training

We recruited and trained over 20 undergraduate students for the learning facilitator role during the study period. Students were interviewed and selected based on their experience and/or willingness to work with children and families, availability to meet consistently twice a week with their assigned participants *via* Zoom, and enthusiasm for the research. Our research team was ethnically and racially diverse, and many students were fluent in languages in addition to English. While over the summer most of the undergraduate students had full-time roles on the project, once the school year began, they had to juggle their work with their own courses and other responsibilities. Given the pandemic, many of the undergraduate students were not living on campus and completed their work from their own homes across the United States and in other countries. Our study team included many first-year students, students working in a lab for the first time, and students who did not come from a science background.

Learning facilitators underwent extensive training before being matched with participants to ensure implementation fidelity. First, we provided training in human subjects research, general strategies for working with young students, and background literature on language/reading and summer interventions. Learning facilitators were also trained on the Learning Ally audiobook platform, and began reading the books used in our study. Because all of these training sessions were remote, learning facilitators could refer back to the recordings as needed. Next, we reviewed the scripts for each lesson with learning facilitators in group meetings. Learning facilitators paired up to practice each component of the lesson with each other (e.g., check in, vocabulary instruction, and scaffolding instruction). Each learning facilitator then recorded a full practice session which was reviewed by a member of the core research team. Learning facilitators received feedback on their recorded session, and those that required additional practice were asked to record new verification videos that implemented this feedback before being assigned participants. Undergraduate students who joined our team after the first summer were matched up with an experienced learning facilitator who served as a mentor and practice partner during training and beyond.

Crucially, training did not cease when learning facilitators began working with participants. All learning facilitators attended weekly meetings where they discussed their participants’ progress and troubleshooted any issues. These issues ranged from how to properly implement specific strategies in the scaffolding curriculum, to how to communicate effectively with caregivers about scheduling, to how to respond to a child that shares difficult personal circumstances (see “Child Disclosure” section, below). Learning facilitators were encouraged to reach out to members of the research team any time they wanted to review a session and discuss strategies for working with a specific child, which was facilitated by the online nature of the study. A member of the research team also spot-checked session videos and provided feedback to learning facilitators as needed to ensure intervention fidelity. Finally, we cultivated an active community in a Slack channel, which allowed learning facilitators to post and answer questions promptly. This multi-tiered network of support enabled our team of undergraduates to thrive in the remote research setting. Notably, in addition to all of their responsibilities as learning facilitators, undergraduates also filled numerous other roles on the project such as developing proximal assessment materials, transcribing language samples, communicating with caregivers, and assisting with data maintenance.

### Online Intervention

#### Technical Challenges During Scaffolding Sessions

The biweekly scaffolding sessions over Zoom introduced challenges unique to the virtual setting. First, researchers were dependent on the capabilities of their own and the participant’s internet connection and thus had to flexibly adapt when the connection was impaired. Many participants occasionally could not see or hear their learning facilitator during crucial parts of the session, or the learning facilitator could not discern what the participant was saying from the lagging audio. Learning facilitators took many steps to troubleshoot these issues while staying on Zoom. Turning cameras off, relocating closer to the Wi-Fi router, asking for a school-provided hotspot, and even using FaceTime or phone calls in tandem with Zoom helped mediate these issues. In a few cases, learning facilitators sent the session’s materials to families ahead of time to print out or download so the child would not have to wait for webpages or screen-sharing to load. Learning facilitators also supported participants who had difficulty logging into or using the Learning Ally audiobook app by asking participants to share their screens and walking them through the setup.

The online setting also enabled children to multitask during sessions. For example, there were numerous instances of participants attending sessions while siblings played video games in the same room, while friends were over, or while simultaneously doing something else on the computer. To address these distractions, learning facilitators would ask, “Are you distracted right now? How can we fix that?” and have the child come up with potential solutions. These solutions included putting on headphones, moving to another room, or asking the people around them to quiet down.

#### Child Disclosure

Disclosure of sensitive information occasionally came up during the testing and scaffolding sessions. In some cases this was prompted, as our study included parent and child questionnaires about experiences during the COVID-19 pandemic, negative feelings, and anxiety/depression. For example, a child disclosed that they thought about death ‘‘all the time’’ in response to a questionnaire item. We also anticipated that some scores on child self-report and parent-report anxiety/depression measures might fall in the clinically elevated range. In other cases, unprompted sensitive information was shared with researchers. For example, one child, when asked to use the vocabulary word ‘evasive’ in a sentence, said that they ‘‘used evasive action to avoid their mother hitting them.’’ To address these expected and unexpected issues, we developed a detailed protocol for the research team to follow, overseen by a clinical psychologist who is a member of the research team. The psychologist checked the questionnaire data for red-flag indicators (supplemental protocol^5^) weekly. If there were indicators that met our criteria for concern (e.g., anxiety or depression scores that were in the clinically elevated range), she reviewed the pertinent data available and contacted the parents/guardians to alert them about the areas of concern and potentially suggest that they consider seeking a professional consultation for further guidance, if they had not already done so. In most cases, the parents/guardians were aware that their child was struggling emotionally (and many had already sought professional help or were in the process of doing so).

If a child indicated negative thoughts or feelings directly to a research team member during a session, the research team members were instructed to notify the psychologist immediately following the session. The psychologist would then follow up with the parents/guardians as necessary. We handled the incidence when a child came up with an example sentence about trying to avoid being hit by their parent differently. Although the role of researchers in mandatory reporting is debated, many states mandate researchers working with children to report suspicion of child abuse (Allen, 2009). Consequently, we called State Child and Family Services, where the family lives, and did an anonymous screening. Based on the information we provided, we were told that “it doesn’t rise to the level of report.” We continued to monitor the child, but nothing alarming came up during the subsequent sessions.

We learned from this study that particularly when frequently working with children directly in their homes or when collecting sensitive information, issues related to children’s safety and wellbeing are likely to come up. We were fortunate to have a trained psychologist on our team who helped us develop a detailed protocol for dealing with these issues and who was responsible for communicating this information to families in a non-alarming but informative manner. Although not always mandated by the IRB, every study that involves children should include detailed procedures for handling sensitive information. Additionally, particularly for online studies that span several states, it is important to know which agency handles suspicions of potential abuse or neglect and what responsibilities researchers working with children have in that state.

Finally, it is important to support team members who may hear from children about difficult challenges they are facing. Most research assistants do not have mental health training, and thus may experience stress or other reactions to instances of child disclosure. Our learning facilitators were undergraduate students who themselves had been dealing with unprecedented challenges related to the pandemic. We addressed these potential challenges explicitly during training and through encouraging continuous communication within the team throughout the study, and by clearly indicating who to contact if such an issue arose. On our Slack channel and during weekly meetings, team members shared their experiences, debriefed, and coached each other on how to best respond to participants. In specific instances (described below), the clinical psychologist on our team provided one-on-one support to team members.

### Qualitative Caregiver and Child Experiences

#### Child Reflections

At the end of the 8 weeks of meeting with learning facilitators, many participants did not want the study to end. When one learning facilitator started the last session with her student by saying, “Are you ready for our last lesson today?” the participant responded, “Yes, but I don’t want it to be our last lesson,” and ended up signing off the call by saying, “Okay, love you, see you, bye!” Another participant who always brought his favorite stuffed animal, Teddy, to the sessions remarked that, “Teddy is sad,” when saying their goodbyes at their final meeting.

Many children reported enjoying the study experience, even if they did not enjoy their regular school-related activities or reading. During her final session, one student remarked “I hate school! School is evil.” The learning facilitator said “Well, this is like school and this was really fun!” to which the participant said, “This wasn’t evil.” One participant who had previously stated he did not enjoy reading told his parent at the 7-week mark: “You know what’s so great about the audiobooks mum? It’s that they’re able to go into such more details than movies!” The parent expanded on this: “I cannot express the joy it brings me to hear my son starting conversations with me about stories he’s read. Last week he wanted to recount some various storylines to me from books. To [say] that we’ve been enjoying the experience is an understatement. Thank you.”

Many children also faced pandemic-related challenges that affected them during the course of the study. In addition to being out of school and having their social lives change, a few had family members who were directly affected by the virus. For instance, one participant was living with an uncle who had COVID-19. During one session, she told her learning facilitator, “People are in my house and it’s difficult for me and my mom because, you know, my uncle is going to die. They want to help him, but they can’t.” One week later, during the routine check-in, the learning facilitator asked how she was doing and the participant said she was sad; “Yesterday, my uncle died. We saw him and, like, it’s sad for me since I [have known] him since I was a kid. Me and my mom [were] crying.” Her learning facilitator expressed her condolences, letting the child know that this is an extremely difficult time. She made sure to offer the participant an opportunity for breaks, instating a codeword of “rainbow sunshine.” The learning facilitators adapted to meet the participants where they were at emotionally and mentally each session, knowing that the pandemic affected everyone’s lives differently, and were generally a welcoming, consistent presence in the participants’ lives for the duration of the study. Importantly, children participating in our research always come into our sessions with a variety of experiences. While the pandemic led to more consistent challenges among our participants, these difficult experiences – death, illness, stress, financial insecurity – should always be on the research team’s radar. At the end of the study, participants in the Scaffolding Group reported generally positive experiences (Table 6).

**TABLE 6:** Child experiences in scaffolding group.

How much did you like meeting with your learning facilitator?	
Not at all	1 (1.8%)
A little bit	3 (5.3%)
Sometimes	11 (19.3%)
A lot	42 (73.7%)
**How often did you feel like you learned new words with your learning facilitator?**	
Not at all	1 (1.8%)
A little bit	5 (8.8%)
Sometimes	11 (19.3%)
A lot	40 (70.2%)

#### Caregiver Reflections

At the end of the study, caregivers filled out a reflection survey about their experience in the study. In general, caregivers of children in the Scaffolding Group did not find it difficult for their child to have biweekly online meetings with their learning facilitator (Table 7).

**TABLE 7:** Caregiver experiences in scaffolding group.

Was it challenging to get your child to meet with their learning facilitator?	
Not at all	50 (80.6%)
A little bit	9 (14.5%)
Sometimes	3 (4.8%)
A lot	0

Caregivers in both the Scaffolding Group and Audiobooks-only group likewise provided open-ended responses about their experiences in the study. Selected representative responses are included below (Table 8). Participants in the Audiobooks-only condition did not meet regularly with a learning facilitator, but they did receive weekly messages with updates on reading milestones and suggested book titles to read.

**TABLE 8:** Caregiver experiences in scaffolding and audiobooks-only groups.

	Scaffolding group	Audiobooks-only group
What did your child enjoy most in this study?	“My child enjoyed all aspects of the study. He is proud to tell others that he is participating in a study. He is very excited to be paid by gift certificates. He loves how he can access any book of his choosing. He enjoyed the experience of meeting weekly and discussing the books with someone.” “My son really enjoyed meeting with the learning facilitator and was sad to learn he would not be meeting with the facilitator anymore. He loved the books and the platform though I was hoping he would read more without me reminding him.” “He enjoyed being introduced to books he may not have otherwise picked out to read. He also liked meeting with his facilitator. He is a social kid and the pandemic has been hard, so seeing [his learning facilitator] was a highlight of the week.”	“He definitely enjoyed listening to the books that were recommended the best!!” “It allowed her to be independent with her nightly reading.” “She enjoyed engaging with the tester. She enjoyed being able to pick her own book and listen on her own. This contributed to family conversations regarding the stories she listened too.” “He really enjoyed the interviews and listening to/reading along w/Learning Ally. I would like to continue it. He would often have siblings gathered around, reading too.” “Es una experiencia bonita para los niños,por que es una manera de leer sin leer osea escuchando, es diferente pero me gusta, hasta la niña de segundo grado quería escuchar los libros, me gusto mucho.gracias sigan asi ayudando a niños a que le den importancia a la lectura.” *Translation: “It is a beautiful experience for the kids because this way they can read with listening, it’s different but I like it. Even my second grade daughter wanted to listen to the books. I enjoyed it a lot. Keep up the good work”*
What did your child find most challenging in this study?	“She found the questions and vocabulary hard.” “He is not used to listening to books and using the app required more setup time since he had to use his laptop, so it was something we had to remind him to do.” “Finding time to read the books, especially without distraction” “Twice weekly meetings with the facilitator was a lot for our schedule” “She sometimes did not want to stop what she was doing to attend scaffolding. Also wanted to socialize and share other things with Facilitator not fully focused on session”	“She did not like listening to books she had no interest in.” “Trying to read/listen to the books she was not immediately interested in. I challenged her to try at least half of the book to see if it improved and she did not like that.” “The second book didn’t hold her interest” “Mostly technical problems” “Por las circunstancias pasa mucho tiempo conectado a algún dispositivo electrónico y aveces solo quería hacer otra cosa, en circunstancias normales creo seria su actividad favorita.” *Translation: “Because of the circumstances he spent a lot of time connected to an electronic device and sometimes he wanted to do something else. Under normal circumstances this might have been his favorite activity”*

As reflected in these responses, caregivers in both groups had many positive experiences in the study. The remote learning environment fostered feelings of social isolation and loneliness for many children (as reflected in our surveys). In the Scaffolding Group, caregivers generally commented on interactions with the learning facilitators, and suggested that the connections forged between children and learning facilitators in our study may have helped ameliorate some of the negative socio-emotional consequences of the pandemic. This positive feedback is useful as we consider implementing future online interventions. In the Audiobooks-only Group, positive feedback focused on the reading experience and book selection.

Challenges were modest for both groups, and some challenges were not unique to the remote nature of the study. For instance, caregivers of children in the Scaffolding Group reported some difficulty finding time for sessions and getting their child to read the books, and some caregivers commented on the challenging nature of the vocabulary. In the Audiobooks-only Group, some caregivers noted that their child was not always interested in the recommended books. This group received the same book recommendations as the Scaffolding Group, but they did not discuss the books with a learning facilitator, which we hypothesized would impact their engagement. The Audiobooks-only Group also received only weekly updates; thus, they were unable to change books that did not interest them as easily as participants in the Scaffolding Group. Some caregivers also reported technical difficulties during and after the study. We relayed all technical issues to the audiobook company, and they worked with us and the caregivers to find solutions.

### Takeaways

To properly measure intervention effects, we needed to ensure that both participants and learning facilitators were properly supported for an online intervention. Particularly for our learning facilitators, who had no previous experience implementing interventions, extensive training and open communication with supervisors and peers was critical. We found that weekly meetings and an internal study Slack channel provided opportunities for learning facilitators to learn from one another and troubleshoot issues. Consistent communication and chances to check-in were crucial since we could not share a physical lab space. Video recording of all sessions allowed for ensuring fidelity of implementation and consistency across different learning facilitators and sessions.

The Scaffolding Group provided useful lessons for other researchers conducting studies with frequent online meetings. Researchers should expect some sessions to have distractions and technical difficulties; thus, it is important to have plans in place to ensure the fidelity of the study. Families reported only modest difficulties with study demands, and feedback from caregivers and children were overall positive. Indeed, many children felt comfortable sharing even highly personal information with their learning facilitators. Researchers should establish clear protocols for how to deal with sensitive information shared by children and families, particularly for studies that involve lots of online interactions.

## Discussion

**FIGURE 5:**
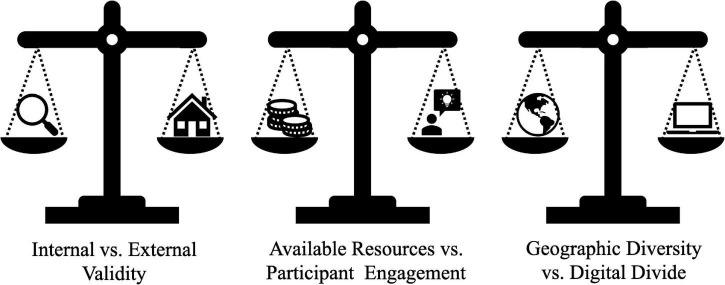
Tradeoffs for online intervention studies with developmental populations.

We implemented a fully remote RCT intervention (final *N* = 255 third and fourth graders, ages 8–10 years) targeting children’s language comprehension skills, which we described as a case study to explore various factors involved in conducting an online intervention study. We have summarized the challenges we faced, solutions we devised, and considerations for future research. Although our project represents a specific case study, and the implications should be considered carefully, we believe that the unique context of our study, its intensity and scale, and our diverse recruitment efforts allow us to derive ‘lessons learned’ that could be useful for others embarking on a similar project. We conclude by discussing what we believe to be the three main tradeoffs to think about when deciding whether and how to implement an online intervention study with a developmental sample (Figure 5).

### Internal vs. External Validity

An important goal of RCTs is to design and evaluate carefully controlled interventions that allow researchers to understand the precise causal mechanisms by which an intervention leads to learning gains. However, this can come at a cost – sometimes, the more controlled the intervention, the less likely it is to work in the “real world.” As with any other type of study, an online RCT intervention requires researchers to consider tradeoffs between internal validity (how well the experiment tests what it is meant to test and is not influenced by other factors) and external validity (how well the experiment replicates in a natural environment).

Most developmental studies optimize internal validity by conducting studies in labs. These studies are well-poised to isolate the precise mechanism or phenomenon researchers are interested in studying. However, there are also drawbacks to in-lab studies that are particularly relevant for researchers interested in conducting RCTs. In-lab developmental studies typically rely on convenience samples, which tend to be homogenous, thereby limiting generalization to other populations (Bornstein et al., 2013). Furthermore, due to multiple practical considerations (e.g., space limitations, transportation, scheduling issues), in-person studies tend to have smaller sample sizes than what is possible in online data collection. Finally, the ecological validity of such studies has been criticized – and the implications for what developmental processes look like in messy and unpredictable real-world settings, such as learning in a child’s home, are limited (Lortie-Forgues and Inglis, 2019). Thus, while implementing an RCT study online in children’s homes requires giving up some of the control of in-lab experiments and introduces additional noise, the tradeoff is that these studies can be more naturalistic and lead to increased sample diversity.

Especially important to consider for intervention studies is generalizability of effectiveness. On the other side of the spectrum from carefully controlled in-lab studies are large-scale educational RCT studies that implement interventions in schools and childcare settings. These studies tend to have higher external validity, but a side effect is increased noise. These studies often build on pilot studies that establish the value of a particular intervention under tightly controlled conditions, but they tend to have small efficacy in these real-world settings (Lortie-Forgues and Inglis, 2019). There are many reasons for this. For example, school settings may be prohibitive of careful sample selection using stringent exclusion criteria (i.e., one child in a classroom receives the intervention while another child does not). Although there are design and statistical methods to overcome these issues (e.g., Regression Discontinuity Design; Lee and Munk, 2008), online intervention studies can bypass them altogether by working with eligible children in their own homes, which expands the pool of participants who are eligible to participate while also allowing the use of specific eligibility criteria and random group assignments. Similarly, it is more difficult to monitor and ensure implementation fidelity of programs when working in complex formal institutional environments such as schools, as compared to negotiating logistics with a child-researcher duo. In our study, we were able to overcome these obstacles because we could closely monitor research activities *via* direct and continuous communication and video recording, and to document possible threats to validity during the various aspects of the study (e.g., background noise, child distraction, connectivity issues, implementation fidelity, etc.).

Thus, we suggest that the online implementation of intervention studies could improve the internal validity of such studies while maintaining their external validity. In online studies, the research team can operate within a well-controlled lab environment, while working with participants in natural, ecologically valid settings. We discussed several potential threats to the validity of our study, such as background noise and technological challenges that could impact reliable data collection. Based on the comparison of the reliability scores for the current study and in-lab studies, however, online data collection resulted in equally reliable data collection, supporting the feasibility of maintaining internal validity in remote developmental research. The increased racial and socioeconomic diversity of the current sample, as compared to in-lab samples, suggests that we were able to achieve greater ecological validity. Furthermore, our study was conducted entirely in children’s natural context – in their own homes – supporting its potential efficacy in real-world settings.

### Available Research Resources vs. Participant Engagement

Implementing an RCT can be resource intensive – e.g., researchers’ time, project budget, number of personnel – and often requires making decisions regarding how many resources to devote in order to maximize participant engagement and retention. Participant engagement can be measured across different levels (Matthews et al., 2011). Recruitment is one such measure that considers the reach of the study to the target population. Many educational intervention studies rely on school partnerships for recruitment, which can be an effective strategy for recruiting a large number of children from diverse educational environments. However, establishing school partnerships requires substantial time and energy. The research team first has to clearly communicate the goals of the intervention and the benefits to that school’s community in order to get buy-in from school leaders and educators. This process typically relies on existing relationships with schools and institutional familiarity, which might be more difficult for a new investigator to establish. Even when schools are interested in a potential partnership, the bureaucratic processes can be extensive before the study can get started. It can also be difficult to randomly assign students to conditions within a school because once a school is enthusiastic about an intervention, the school often wants all their students to be placed in the intervention condition.

On the other hand, many developmental science studies recruit participants directly through advertisements and social media (Hurwitz et al., 2017). Social media recruitment efforts can reach a wide pool of potential participants at a reasonably low cost. Our social media reach was extensive, reaching people from hundreds of different zip codes across the United States, but this required intentional targeted advertising. Based on our recruitment data, through school partnerships and social media, we successfully reached the participant demographic we set to recruit.

Enrollment, retention, and intervention adherence are additional types of engagement, each with its own set of challenges. Our enrollment and retention outcomes were less successful than our recruitment reach. Our final sample, although still very diverse, was not representative of the diversity in schools and communities we targeted in our recruitment. For example, household income eligibility for free/reduced lunch is around $52,000. Although we targeted schools and communities with a high proportion of free/reduced lunch eligibility, we ended up with a median income with the $80,000–120,000 range. Thus, even though we allocated almost all of our recruiting budget and efforts to recruit lower-SES participants, our final enrollment was not skewed toward this demographic. Retention and intervention adherence represent two of the most critical factors to ensure the validity of intervention studies (Slack and Draugalis, 2001) and are most difficult to achieve when working with disadvantaged communities. Ensuring participant engagement in such communities is resource-intensive, requiring a substantial recruitment budget, a large and well-trained research team, and attractive incentives for participation.

There is a large body of evidence from parenting programs targeting underserved communities that show how program-level factors (e.g., team member composition, level of family support provided) interact with participant factors (e.g., SES, job demands, perception of research, language barriers) in ensuring enrollment and retention (Whittaker and Cowley, 2012; Hackworth et al., 2018). Families, especially those from lower-SES backgrounds, are more likely to enroll and stay in a program, for example, if they have an experienced research liaison who supports them in identifying and overcoming barriers to participation (Rivas-Drake et al., 2016; Hackworth et al., 2018). Our full-time, bilingual coordinators were available to check in and assist families using preferred communication methods, and researchers assisted families with troubleshooting the apps for the intervention. Clear communication on research objectives and the theoretical foundation of the intervention is important for reducing perceptual barriers to participation (Barlow et al., 2003; Moran et al., 2004). Professionalism and experience of team members (Hackworth et al., 2018), as well as their representativeness of the target community (Gray, 2002), were additional factors that ensured engagement. During our consent process, as well throughout the study, researchers were available to answer questions. We also hosted several information sessions for teachers and administrators in our partner district, as well as a bilingual (Spanish/English) session for parents at one of our partner schools. Intervention effects have been more significant in well-resourced studies, as compared to studies with fewer resources (Kim and Quinn, 2013). In general, across studies, there is an agreement that intervention programs targeting lower-SES communities require careful considerations of various factors that could affect direction of resources toward alleviating these barriers.

Online research may seem like a low-resource opportunity for obtaining larger, more diverse samples. With the advent of online platforms for developmental studies (e.g., Discoveries Online; Lookit), unmoderated research studies have become increasingly popular. Such studies, which allow participants to complete tasks on their own time and without the researcher’s direct involvement, front-load their resources for design but require minimal resources for implementation. We caution, however, that families from underrepresented backgrounds may still face greater barriers to engaging in such studies than participants that are typically included in research studies, and we echo calls to actively work toward providing support and internet access for these populations (Lourenco and Tasimi, 2020; Sheskin et al., 2020). This is particularly pertinent for longitudinal and intervention studies that require substantial researcher moderation in order to be successful. Indeed, a similar online intervention during the pandemic that did not explicitly target a diverse sample based on SES ended up with almost all mothers with at least a 4-year college degree (Bambha and Casasola, 2021). We found that even children in school systems that did provide devices and internet access sometimes experienced technical difficulties in our study. Thus, while online RCTs can remove certain resource constraints (such as space and travel compensation), researchers should expect to invest significant time and effort to achieve diverse samples and ensure their participation.

### Geographic Diversity vs. Digital Divide

Online study participation with children, although not always feasible, can significantly increase sample diversity by allowing easy access regardless of a family’s geographic location and by minimizing caregivers’ time commitment (Rhodes et al., 2020; Sheskin et al., 2020). This is particularly crucial for longitudinal studies that include multiple sessions and a significant time commitment. Online developmental studies have recruited more diverse samples than in-lab developmental studies (e.g., Scott and Schulz, 2017; Scott et al., 2017), including more geographically diverse samples (Bambha and Casasola, 2021). Our study recruited participants from 26 different states in the United States (Figure 2), and our sample was comparable to or better than our prior in-lab studies in terms of socioeconomic and racial diversity (Figure 1). However, the accessibility of online study participation is still challenging for many families (Lourenco and Tasimi, 2020). Prior to the start of the pandemic, almost a third of public K-12 students in the United States lacked adequate internet access and/or an adequate device for distance learning (Chandra et al., 2020). While some school systems provided children with computers and internet access to enable remote learning, many children still lack technology that would enable them to participate in an online intervention study. We unfortunately had to exclude interested families who lacked a computer or tablet at home due to our assessment battery. Furthermore, the “digital divide” – that is, the gap between people who have computer and internet access and those who do not – is not equally distributed across geographic boundaries and demographic groups (Van Dijk, 2020). 37% of students in rural communities in the United States lack adequate internet connectivity at home, compared to 21% of students in urban environments (Chandra et al., 2020). Many of our participants struggled with internet connectivity issues and other technological challenges over the course of the study. Thus, it is important to take into account not only whether participants have access, but also whether they have complete access to these studies. In contrast, intervention studies that do not require the family to learn about the study and participate through their own technological platforms (such as most in-school interventions) allow researchers to ensure all participants in a constrained location can participate. Yet in-person interventions are not equally accessible to all geographic regions either – most of these studies take place near research institutions. One solution is to provide participants with the technology they need to participate in online research studies (Lourenco and Tasimi, 2020). Though adding additional costs to the study budget, providing devices with mobile data may lead to more representative samples as well as better data quality. For example, several large-scale projects have successfully deployed mobile devices loaded with educational content in rural locations in the United States and around the world, like small villages in Africa (Breazeal et al., 2016; Uchidiuno et al., 2018). This tradeoff may be worth the cost, particularly for home-based intervention studies. Online studies allow for geographic diversity of the research team as well. Our study team worked from multiple time zones, which allowed us to accommodate participants from across the United States. This also opens up the possibility for recruiting community members to be part of the research team. This type of participatory research may lead to higher recruitment, retention, and validity of intervention studies (Levac et al., 2019).

## Conclusion

In response to the COVID-19 pandemic we conducted a scalable online RCT intervention study with children from diverse backgrounds across the United States. In this paper, we summarized the challenges we encountered and the tradeoffs to consider when implementing such studies. Despite possible threats to the internal validity of our study, difficulties in reaching demographically diverse populations, and resource-exhaustive efforts to support participant engagement and retention, we were able to conduct a study that provided educational support during a challenging time for both children and their caregivers. With the aforementioned considerations and tradeoffs in mind, we believe that fully remote intervention studies are a worthwhile endeavor for developmental researchers, and we expect to see more of them in the future.

## Data Availability Statement

The datasets presented in this study can be found in online repositories. The names of the repository/repositories and accession number(s) can be found below: OSF: https://osf.io/zac9d/.

## Ethics Statement

Studies involving human participants were reviewed and approved by the Committee on the Use of Human Experimental Subjects at MIT. Informed consent to participate in this study was provided by the participants’ parents or legal guardians.

## Author Contributions

OO-P and HO contributed equally to the study idea, design, implementation, and manuscript preparation. XA and HK assisted with study design. XA, HK, JS-F, KW, YCT, NG, and JD assisted with study implementation, and each contributed to writing one or more sections and designed figures for this manuscript. JG contributed to the study idea and design, and provided feedback on this manuscript. All authors contributed to the article and approved the submitted version.

## Conflict of Interest

The authors declare that the research was conducted in the absence of any commercial or financial relationships that could be construed as a potential conflict of interest. The reviewer JFK declared a shared affiliation, with one of the authors OO-P to the handling editor at the time of the review.

## Publisher’s Note

All claims expressed in this article are solely those of the authors and do not necessarily represent those of their affiliated organizations, or those of the publisher, the editors and the reviewers. Any product that may be evaluated in this article, or claim that may be made by its manufacturer, is not guaranteed or endorsed by the publisher.

## Footnotes

1
https://childrenhelpingscience.com

2
https://lookit.mit.edu

3
https://learningally.org/

4 Olson, H. A., Ozernov-Palchik, O., Arechiga, X. M., Wang, K. L., and Gabrieli, J. D. E. (in preparation). Effects of remote voluntary audiobook randomized controlled trial intervention on children s language skills. *Manuscript in Preparation.*

5
https://osf.io/6urmx/

## References

[B1] Allen B. (2009). Are researchers ethically obligated to report suspected child maltreatment? a critical analysis of opposing perspectives. *Ethic. Behav.* 19 15–24. 10.1080/10508420802623641

[B2] Bambha V. P. Casasola M. (2021). From Lab to Zoom: Adapting training study methodologies to remote conditions. *Front. Psychol.* 2021:12. 10.3389/fpsyg.2021.694728 34349707 PMC8326395

[B3] Barlow J. Coren E. Stewart-Brown S. (2003). Parent-training programmes for improving maternal psychosocial health. *Coch. Datab. Syst. Rev.* 2003:4. 10.1002/14651858.CD002020 11034741

[B4] Barton E. E. Fettig A. (2013). Parent-implemented interventions for young children with disabilities: a review of fidelity features. *J. Early Interv.* 35 194–219.

[B5] Bornstein M. H. Jager J. Putnick D. L. (2013). Sampling in developmental science: Situations, shortcomings, solutions, and standards. *Dev. Rev.* 33 357–370. 10.1016/j.dr.2013.08.003 25580049 PMC4286359

[B6] Breazeal C. Morris R. Gottwald S. Galyean T. Wolf M. (2016). Mobile devices for early literacy intervention and research with global reach. *ACM Conf.* 2016 11–20. 10.1002/cad.20225 29243382

[B7] Casler K. Bickel L. Hackett E. (2013). Separate but equal? A comparison of participants and data gathered *via* Amazon’s MTurk, social media, and face-to-face behavioral testing. *Comp. Hum. Behav.* 29 2156–2160.

[B8] Chandra S. Chang A. Day L. Fazlullah A. Liu J. McBride L. (2020). *Closing the K–12 digital divide in the age of distance learning.* Boston, MA: Common Sense and Boston Consulting Group.

[B9] Chuey A. Asaba M. Bridgers S. Carrillo B. Dietz G. Garcia T. (2021). Moderated online data-collection for developmental research: methods and replications. *Front. Psychol.* 12:734398. 10.3389/fpsyg.2021.734398 34803813 PMC8595939

[B10] Dunn L. M. Dunn D. M. (2007). *Peabody picture vocabulary test–fourth edition (PPVT-4).* Circle Pines, MN: AGS.

[B11] Fixen D. Naoom S. Blase K. Friedman R. Wallace F. (2005). *Implementation Research: A Synthesis of the Literature.* Tampa: University of South Florida The National Implementation Research Network.

[B12] George F. Mallery M. (2003). *Quantitative method for estimating the reliability of data.* Kalamazoo, MI: Western Machinegun University.

[B13] Gillen N. A. Siow S. Lepadatu I. Sucevic J. Plunkett K. Duta M. (2021). Tapping into the potential of remote developmental research: introducing the OxfordBabylab app. *PsyArXiv* 2021:1. 10.1002/9780470773307.ch1

[B14] Good R. H. Kaminski R. A. Smith S. Laimon D. (2002). *Dynamic indicators of basic early literacy skills: DIBELS.* Houston, TX: Dynamic Measurement Group.

[B16] Gray B. (2002). Emotional labour and befriending in family support and child protection in Tower Hamlets. *Child Family Soc. Work* 7 13–22. 10.1046/j.1365-2206.2002.00222.x

[B17] Hackworth N. J. Matthews J. Westrupp E. M. Nguyen C. Phan T. Scicluna A. (2018). What influences parental engagement in early intervention? Parent, program and community predictors of enrolment, retention and involvement. *Prev. Sci.* 19 880–893. 10.1007/s11121-018-0897-2 29629506 PMC6182377

[B18] Harris P. A. Taylor R. Minor B. L. Elliott V. Fernandez M. O’Neal L. (2019). The REDCap consortium: Building an international community of software platform partners. *J. Biomed. Inform.* 95:103208. 10.1016/j.jbi.2019.103208 31078660 PMC7254481

[B19] Harris P. A. Taylor R. Thielke R. Payne J. Gonzalez N. Conde J. G. (2009). Research electronic data capture (REDCap)—A metadata-driven methodology and workflow process for providing translational research informatics support. *J. Biomed. Inform.* 42 377–381. 10.1016/j.jbi.2008.08.010 18929686 PMC2700030

[B20] Heinrichs N. Bertram H. Kuschel A. Hahlweg K. (2005). Parent recruitment and retention in a universal prevention program for child behavior and emotional problems: Barriers to research and program participation. *Prevent. Sci.* 6 275–286. 10.1007/s11121-005-0006-1 16075192

[B21] Hurwitz L. B. Schmitt K. L. Olsen M. K. (2017). Facilitating development research: Suggestions for recruiting and re-recruiting children and families. *Front. Psychol.* 8:1525. 10.3389/fpsyg.2017.01525 28955265 PMC5601975

[B22] Jiang H. Logan J. (2019). Improving reading comprehension in the primary grades: mediated effects of a language-focused classroom intervention. *J. Speech Lang. Hear. Res.* 62 2812–2828. 10.1044/2019_JSLHR-L-19-0015 31390289

[B23] Kaufman A. S. (2004). *Kaufman brief intelligence test–second edition (KBIT-2).* Circle Pines, MN: American Guidance Service.

[B24] Kim J. S. Quinn D. M. (2013). The effects of summer reading on low-income children’s literacy achievement from kindergarten to grade 8: A meta-analysis of classroom and home interventions. *Rev. Educ. Res.* 83 386–431.

[B25] Kizilcec R. F. Reich J. Yeomans M. Dann C. Brunskill E. Lopez G. (2020). Scaling up behavioral science interventions in online education. *Proc. Natl. Acad. Sci.* 117 14900–14905. 10.1073/pnas.1921417117 32541050 PMC7334459

[B26] Kraft M. A. (2020). Interpreting effect sizes of education interventions. *Educ. Res.* 49 241–253. 10.3102/0013189x20912798

[B27] Lee H. Munk T. (2008). *Using regression discontinuity design for program evaluation.* Rockville, MD: Research Blvd, 3–7.

[B28] Levac L. Ronis S. Cowper-Smith Y. Vaccarino O. (2019). A scoping review: The utility of participatory research approaches in psychology. *J. Comm. Psychol.* 47 1865–1892. 10.1002/jcop.22231 31441516 PMC6852237

[B29] Lingwood J. Levy R. Billington J. Rowland C. (2020). Barriers and solutions to participation in family-based education interventions. *Internat. J. Soc. Res. Method.* 23 185–198. 10.1080/13645579.2019.1645377

[B30] Lorenc T. Petticrew M. Welch V. Tugwell P. (2013). What types of interventions generate inequalities? Evidence from systematic reviews. *J. Epidemiol. Comm. Health* 67 190–193. 10.1136/jech-2012-201257 22875078

[B31] Lortie-Forgues H. Inglis M. (2019). Rigorous large-scale educational RCTs are often uninformative: Should we be concerned? *Educ. Res.* 48 158–166.

[B32] Lourenco S. F. Tasimi A. (2020). No participant left behind: Conducting science during COVID-19. *Trends Cogn. Sci.* 24 583–584. 10.1016/j.tics.2020.05.003 32451239 PMC7211671

[B33] Manz P. H. Hughes C. Barnabas E. Bracaliello C. Ginsburg-Block M. (2010). A descriptive review and meta-analysis of family-based emergent literacy interventions: To what extent is the research applicable to low-income, ethnic-minority or linguistically-diverse young children? *Early Childh. Res. Q.* 25 409–431.

[B34] Matthews E. C. Fox S. Hackworth N. Kitanovski M. Vista A. (2011). *The Parenting Research Centre. We wish to acknowledge the valuable contributions and support of: Iris Crook, Community Development Worker, Family & Children’s Services, Yarra Ranges Shire; Georgina Devereaux, Playgroup Support and Development Officer, Frankston City Council.* Victoria: Partnerships Division Office for Children and Portfolio Coordination Department of Education and Early Childhood Development Melbourne.

[B35] Moran P. Ghate D. Van Der Merwe A., and Policy Research Bureau (2004). *What works in parenting support?: A review of the international evidence.* London: DfES Publications.

[B36] Nicholson L. M. Schwirian P. M. Klein E. G. Skybo T. Murray-Johnson L. Eneli I. (2011). Recruitment and retention strategies in longitudinal clinical studies with low-income populations. *Contemp. Clin. Trials* 32 353–362. 10.1016/j.cct.2011.01.007 21276876 PMC3070062

[B37] Nielsen M. Haun D. Kärtner J. Legare C. H. (2017). The persistent sampling bias in developmental psychology: A call to action. *J. Exp. Child Psychol.* 162 31–38. 10.1016/j.jecp.2017.04.017 28575664 PMC10675994

[B38] O’Donnell C. L. (2008). Defining, conceptualizing, and measuring fidelity of implementation and its relationship to outcomes in K–12 curriculum intervention research. *Rev. Educ. Res.* 78 33–84.

[B39] Ozernov-Palchik O. Norton E. S. Sideridis G. Beach S. D. Wolf M. Gabrieli J. D. (2017). Longitudinal stability of pre-reading skill profiles of kindergarten children: Implications for early screening and theories of reading. *Dev. Sci.* 20:e12471. 10.1111/desc.12471 27747988 PMC5393968

[B40] Pollack C. Wilmot D. Centanni T. Halverson K. Frosch I. D’Mello A. (2021). Anxiety, motivation, and competence in mathematics and reading for children with and without learning difficulties. *Front. Psychol.* 2021:704821. 10.3389/fpsyg.2021.704821 34690863 PMC8528962

[B41] Rhodes M. Rizzo M. T. Foster-Hanson E. Moty K. Leshin R. A. Wang M. (2020). Advancing developmental science *via* unmoderated remote research with children. *J. Cogn. Dev.* 21 477–493. 10.1080/15248372.2020.1797751 32982602 PMC7513948

[B42] Rivas-Drake D. Camacho T. C. Guillaume C. (2016). Just good developmental science: Trust, identity, and responsibility in ethnic minority recruitment and retention. *Adv. Child Dev. Behav.* 50 161–188. 10.1016/bs.acdb.2015.11.002 26956073

[B43] Scott K. Chu J. Schulz L. (2017). Lookit (Part 2): Assessing the viability of online developmental research, results from three case studies. *Open Mind* 1 15–29. 10.1162/opmi_a_00001

[B44] Scott K. Schulz L. (2017). Lookit (part 1): A new online platform for developmental research. *Open Mind* 1 4–14. 10.1162/opmi_a_00002

[B45] Sheskin M. Scott K. Mills C. M. Bergelson E. Bonawitz E. Spelke E. S. (2020). Online developmental science to foster innovation, access, and impact. *Trends Cogn. Sci.* 24 675–678. 10.1016/j.tics.2020.06.004 32624386 PMC7331515

[B46] Slack M. K. Draugalis J. R. Jr. (2001). Establishing the internal and external validity of experimental studies. *Am. J. Health-Syst. Pharm.* 58 2173–2181. 10.1093/ajhp/58.22.2173 11760921

[B47] Uchidiuno J. Yarzebinski E. Madaio M. Maheshwari N. Koedinger K. Ogan A. (2018). *Designing appropriate learning technologies for school vs home settings in tanzanian rural villages.* Seattle: ACM SIGCAS Conference, 1–11.

[B48] Van Dijk J. (2020). *The digital divide.* Hoboken: John Wiley & Sons.

[B49] Veinot T. C. Mitchell H. Ancker J. S. (2018). Good intentions are not enough: How informatics interventions can worsen inequality. *J. Am. Med. Inform. Assoc.* 25 1080–1088. 10.1093/jamia/ocy052 29788380 PMC7646885

[B50] Wagner R. K. Torgesen J. K. Rashotte C. A. Pearson N. A. (1999). *Comprehensive test of phonological processing: CTOPP.* Austin, TX: Pro-ed inc.

[B51] Wechsler D. (2011). *WASI-II: Wechsler abbreviated scale of intelligence.* London: PsychCorp.

[B52] Whittaker K. A. Cowley S. (2012). An effective programme is not enough: A review of factors associated with poor attendance and engagement with parenting support programmes. *Child. Soc.* 26 138–149. 10.1111/j.1099-0860.2010.00333.x

[B53] Wiig E. H. Secord W. A. Semel E. (2013). *Clinical evaluation of language fundamentals: CELF-5.* San Antonio, TX: Pearson.

